# Editorial: Women in psychiatry 2023: Perinatal psychiatry

**DOI:** 10.3389/fpsyt.2024.1478300

**Published:** 2024-10-07

**Authors:** Soudabeh Givrad, Alison Hermann, Laura Orsolini

**Affiliations:** ^1^ Department of Psychiatry, Weill Cornell Medical Center, New York, NY, United States; ^2^ Unit of Clinical Psychiatry, Department of Experimental and Clinical Neurosciences (DIMSC)/Neurosciences, Polytechnic University of Marche, Ancona, Italy

**Keywords:** perinatal psychiatry, women’s mental health, women’s health, reproductive psychiatry, women researchers

Sex and gender equity in mental health research should be a requisite of a civil and modern society. Mental health professionals, including psychiatrists, should have a social responsibility to advocate for a reduction in social, work, and clinical inequalities for all clinicians and researchers, including those related to sex and gender. An urgent call is needed regarding women’s research priority areas, such as mental health issues throughout the reproductive life cycle. Despite advances in improving representation of women in research careers and publications, they are still underrepresented and underrecognized in leading academic roles, especially in mid and late-career (1). Many factors, such as long-standing biases, gender stereotypes as well as inequity in domestic and caretaking roles outside work, have contributed to women’s underrepresentation in leading psychiatric research roles. The COVID-19 pandemic notably worsened this gap (2). Correcting long-standing inequities in career advancement for women conducting mental health research will require multiple approaches, including better investment in mentoring programs for women and further development of professional organizations to support the work of women researchers (1). Journals publishing mental health research also have an opportunity to advance this effort. In this Research Topic, Frontiers in Psychiatry highlights the work by and for women in psychiatry.

This Research Topic focuses on the work of women researchers in the field of perinatal psychiatry. There have been many clinical, advocacy, research, and educational activities in the field of perinatal psychiatry over the past decade. The perinatal period is a biologically, psychologically, and socially unique time with two-generational implications for health and disease. Perinatal mood and anxiety disorders (PMADs), for instance, are among the most common complications of pregnancy and can negatively impact the health of pregnant women and their offspring(s) and affect pregnancy and neonatal outcomes. Importantly, minorities, individuals in disadvantaged contexts, and those with medically complex pregnancies may experience remarkably higher rates of mental distress during this vulnerable time. As such, there remains a critical need for better research and understanding of biological and psychological processes and associated risk and protective factors, as well as the best models of delivery of clinical care and education for professionals and the general population.

*Women in psychiatry 2023: perinatal psychiatry* addresses these gaps and needs by representing a diverse international body of research in several areas of the field of perinatal psychiatry. A series of studies in this Research Topic focus on investigating risk factors for perinatal mental illness. Fish-Williamson and Hahn-Halbrook conducted a meta-analysis and meta-regression of 412 studies to better explore the association between nutritional factors and cross-national prevalence of postpartum depression (PPD) and, interestingly, found the overall prevalence of PPD to be higher than previously estimated. Furthermore, they found higher rates of PPD in countries with higher consumption of sugar-sweetened beverages. Liu et al. performed an observational research followed by a two-sample Mendelian randomization analysis to explore risk factors for PPD. The authors found a significant association between decreased levels of total bilirubin and the incidence of PPD. Albertini et al. investigated the prevalence of prenatal depression and its associated factors in low- and high-risk prenatal patients using a retrospective and prospective cross-sectional design. The authors found that epilepsy, unfavorable economic conditions, and living without a partner increased the risk of prenatal depression. However, they did not find any association between obstetric risk and the prevalence of prenatal depression, a finding that needs to be further investigated. Taking a more behavioral approach to better elucidate factors associated with PPD, Matsunaga et al. found that increased levels of rumination and decreased exposure to reward perception and behavioral activation were associated with higher rates of PPD, particularly in high-risk groups such as those with a history of mental illness. Their study was conducted during the COVID-19 pandemic, a period that led to increased isolation and rumination in the perinatal population. Therefore, the study showed clinical implications that could guide clinicians in providing perinatal population during large-scale stressful and/or traumatic events leading to isolation or higher levels of anxiety/rumination. Bonanni et al. ascertained whether couples who have twins are overly at higher risk for developing a mental illness by not identifying any significant differences in the psychological well-being among parents of twins and singletons through their retrospective study. Their study was performed in a setting in which mothers of twins automatically received psychological support and, hence, might also be representative of the beneficial role of psychological support. Quiray et al. carried out a qualitative study, using focus groups with doulas and interviews with doula clients, to investigate the role doulas can play in supporting perinatal mental health. Their study showed promising results, in particular, if doulas can be provided with appropriate support and training.

Two articles in this Research Topic focused on the role of artificial intelligence (AI) in perinatal psychiatry. In light of rapid developments in the use of AI in healthcare, Turchioe et al. discussed the importance of responsible AI (RAI) by using a patient-centered approach that is based on principles of autonomy, beneficence, justice, trust, privacy, and transparency. Patra et al. provided findings on natural language processing (NLP), one of the AI branches. Their study showed that NLP can guide patients to high-quality lay reading materials as cost-effective, readily available health education related to pregnancy health and PPD.

Acknowledging race/ethnicity as a social construct that influences health disparities, Beck et al. looked at trends in diagnosed behavioral and substance use disorders in 736,325 deliveries between 2008 and 2020. They identified an increase in the diagnosis of mental health disorders over the years, noting the greatest increase in the Asian population.

PMADs are the most common and best-studied mental disorders during the perinatal period. As such, we need more research elucidating the trajectory of other mental illnesses during this period. Sommerfeldt et al. addressed this need by studying the trajectory of eating disorders during pregnancy and the early postpartum period.

Lastly, Athan broadened the lens beyond traditional psychopathology by discussing psychological, social, cultural, and existential transitions experienced by women who are going to become mothers, known as ‘matrescence’.

Hopefully, this initiative illustrates the excellent and qualified research work led and guided by women researchers in the field of perinatal psychiatry, and encourage future work for and by women scientists.
